# Comparison of Short Vs. Long Half-Life Single-Dose Prophylactic Antibiotics for Cesarean Section

**DOI:** 10.1155/S1064744994000505

**Published:** 1994

**Authors:** Bernard Gonik, James McGregor

**Affiliations:** 1 Department of Obstetrics and Gynecology University of Texas Medical School at Houston Houston TX USA; 2 Department of Obstetrics and Gynecology University of Colorado Health Sciences Center Denver CO USA; 3 Department of Obstetrics and Gynecology Wayne State University School of Medicine Grace Hospital 6071 W. Outer Drive Detroit MI 48235 USA

## Abstract

*Objective:* Numerous studies demonstrate the efficacy of antibiotic prophylaxis for reducing postcesarean section infectious morbidity. The duration of therapy, however, remains controversial. Cost containment measures and the ease of single dosing have led to the introduction of “extended” half-life agents for cesarean-section chemoprophylaxis. We tested the hypothesis that there was no difference in efficacy between a single dose of a short half-life agent (cefoxitin) and a longer half-life agent (cefotetan).

*Methods:* A prospective, double-blind trial of 375 non-elective cesarean-section cases was carried out. Study antibiotics (2 g) were administered intravenously (IV) at cord clamping only.

*Results:* Demographic and clinical variables between the 2 study groups were similar. No significant differences were noted in major or minor morbidity or in infectious morbidities for patients receiving the 2 prophylactic regimens. The occurrence of postoperative endometritis was likewise similar for the subjects receiving cefoxitin (10%) and cefotetan (15%). When cases whose surgery lasted >60 min were evaluated separately, no differences in outcomes between the 2 groups were identified

*Conclusions:* These findings confirm our hypothesis that the half-life difference between these 2 agents does not impact on single-dose Prophylactic efficacy in cesarean section.


Infectious Diseases in Obstetrics and Gynecology 2:120–125 (1994)
© 1994 Wiley-Liss, Inc.

### Comparison of Short Vs. Long Half-Life Single-Dose Prophylactic Antibiotics for Cesarean Section

**Bernard Gonik and James McGregor**

*Department of Obstetrics and Gynecology, University of Texas Medical School at Houston, Houston, TX (B.G.), and Department of Obstetrics and Gynecology, University of Colorado Health Sciences Center, Denver, CO (J.M.)*

ABSTRACT

***Objective:*** **Numerous studies demonstrate the efficacy of antibiotic prophylaxis for reducing post-cesarean section infectious morbidity. The duration of therapy, however, remains controversial. Cost containment measures and the ease of single dosing have led to the introduction of "extended" half-life agents for cesarean-section chemoprophylaxis. We tested the hypothesis that there was no difference in efficacy between a single dose of a short half-life agent (cefoxitin) and a longer half-life agent (cefotetan).**

***Methods:*** **A prospective, double-blind trial of 375 non-elective cesarean-section cases was carried out. Study antibiotics (2 g) were administered intravenously (IV) at cord clamping only.**

***Results:*** **Demographic and clinical variables between the 2 study groups were similar. No significant differences were noted in major or minor morbidity or in infectious morbidities for patients receiving the 2 prophylactic regimens. The occurrence of postoperative endometritis was likewise similar for the subjects receiving cefoxitin (10%) and cefotetan (15%). When cases whose surgery lasted >60 min were evaluated separately, no differences in outcomes between the 2 groups were identified.**

***Conclusions:*** **These findings confirm our hypothesis that the half-life difference between these 2 agents does not impact on single-dose prophylactic efficacy in cesarean section.**
© 1994 Wiley-Liss, Inc.

KEY WORDS
Prophylaxis, antibiotics, infectious morbidity

Numerous studies have demonstrated the efficacy of antibiotic prophylaxis in reducing post-cesarean section infectious morbidity.[1] Controversy regarding the duration of therapy and cost containment measures have led to the introduction of "extended" half-life ($t_{1/2}$) agents for single-dose use. Since, in order to be effective, a prophylactic agent only needs to be present at the time of bacterial contamination,[2] we tested the hypothesis that there was no difference in efficacy between a short half-life agent (cefoxitin: $t_{1/2}$ 41–59 min) and a longer-life agent (cefotetan: $t_{1/2}$ 180–276 min). These 2 agents have a similar antibacterial spectrum and are both recommended to reduce infectious morbidity after cesarean section.[3,4]

### SUBJECTS AND METHODS

A prospective trial was conducted from May 1987 through April 1989 at the University of Texas Medical School at Houston and the University of Colorado Health Sciences Center in Denver involving 375 subjects undergoing non-elective ce-

Address correspondence/reprint requests to Dr. Bernard Gonik, Department of Obstetrics and Gynecology, Wayne State University School of Medicine, Grace Hospital, 6071 W. Outer Drive, Detroit, MI 48235.

Presented at the 33rd Interscience Conference on Antimicrobial Agents and Chemotherapy (ICAAC), Abstract No. 164, New Orleans, LA, October 18, 1993.

Received April 12, 1994
Accepted July 6, 1994

sarean section. This project was approved by each institution's investigational review board, and written consent was obtained from each subject at entry in the study.

Patients who underwent cesarean section with at least 1 major risk factor for developing postoperative infectious morbidity were entered to the study. The risk factors considered for entry were labor, membrane rupture, >3 pelvic examinations, and internal-pressure-transducer monitoring. Patients were also included in the study if, at the discretion of the principal investigator, a minor risk factor (such as obesity, anemia, or intraoperative contamination) mandated antibiotic prophylaxis.

Patients were excluded from the study if they met any of the following criteria: known hypersensitivity to cephamycins or penicillins; active infection or fever at the time of cesarean section; systemic antibiotics within 48 h of surgery; and history of renal or hepatic impairment. This study was a randomized, double-blind comparison of a 2-g single intravenous (IV) dose of cefoxitin and a 2-g single IV dose of cefotetan administered at the time that the umbilical cord was clamped. Prior to entering the study, patients gave complete histories and underwent physical examinations. A complete blood count (CBC), prothrombin time, rapid plasma reagin test (RPR), and routine urinalysis were done. An oral or rectal temperature was recorded. At the time of cesarean section, incision and closure times of the abdomen were recorded and pretherapy aerobic and anaerobic cultures of the hysterotomy site were collected. Culture and sensitivity procedures were run in accordance to NCCLS guidelines.

After surgery, temperature and other vital signs were taken at least every 4 h until discharge from the hospital. In the event of an oral temperature ≥38°C on 2 occasions at least 4 h apart within any 24-h period, excluding the first postoperative day, the following procedures were generally carried out to better characterize this febrile morbidity: physical examination to localize the potential source of fever; chest X-ray if pulmonary pathology was suspected; urinalysis followed by urine culture if suggestive of infection; and CBC with differential. Additional antibiotic therapy was initiated after the above diagnostic studies were completed if deemed necessary by the attending physician.

Judgments of prophylactic efficacy were made by the investigator after the completion of the study-drug therapy prior to unblinding of the study. An assessment of the efficacy was based on the occurrence of major or minor postoperative morbidity. Major morbidity was defined by the occurrence of 1 or more of the following: generalized sepsis (temperature ≥38.8°C with or without bacteremia); endometritis (elevated temperature, abnormally tender uterus, foul-smelling lochia, and absence of other significant pathology); wound infection with dehiscence or requirement for secondary closure; pelvic abscess; pelvic cellulitis; or pelvic thrombophlebitis. Minor morbidity was defined by the occurrence of 1 or more of the following: mild-to-moderate wound infection as defined by purulent drainage from the suture site and cellulitis of the wound; urinary-tract infection; postoperative respiratory infection; patient requiring antibiotics; antipyretics with or without proven infection; febrile morbidity as previously defined without identified etiology.

All patients were evaluated for safety. Clinical adverse experiences were described and recorded by the investigator, who determined the duration, seriousness, severity, and drug relationship as well as the eventual outcome of each adverse experience. Tolerability to the study drug was also addressed. The chi-squared or Fisher exact test was used to compare the 2 treatment groups with respect to patient characteristics, prophylactic success, and incidence of clinical or laboratory adverse experiences. A t-test was used to compare mean ages, gravidity, parity, duration of surgery, duration of postoperative hospitalization, duration of ruptured membranes, duration of labor, and number of vaginal examinations. All tests were 2-tailed and were done at the 5% significance level.

### RESULTS

A total of 375 subjects undergoing cesarean section were enrolled in this study, 189 of whom were treated with a single 2-g IV dose of cefoxitin and 186 of whom were treated with a single 2-g IV dose of cefotetan. Three hundred thirty-seven patients were evaluable for efficacy: 169 in the cefoxitin group and 168 in the cefotetan group. The patient characteristics are summarized in Table 1. The majority of patients (68%) underwent a primary cesarean section. On average, the surgical procedure lasted for <1 h (54 min). The median dura-

TABLE 1. Demographic and clinical variables of study groups[a]

| | Cefoxitin (N = 189) | Cefotetan (N = 186) |
|---|---|---|
| Age (years) | 25.5 ± 5.7 | 25.4 ± 5.7 |
| Race | | |
| Caucasian | 83 | 75 |
| Black | 56 | 59 |
| Other | 50 | 52 |
| Primary cesarean section (No.) | 119* | 137 |
| Gravidity | 2.7 ± 1.8 | 2.4 ± 2.0 |
| Duration of surgery (min) | 55.5 ± 25.9 | 52.9 ± 19.8 |
| Postoperative hospitalization (days) | 4.3 ± 1.8 | 4.7 ± 3.2 |
| Vaginal examinations (No.) | 3.7 ± 2.7** | 4.6 ± 3.2 |
| Median duration of ruptured membranes (h) | 7 | 8 |
| Median duration of labor (h) | 12 | 12 |

[a]Data presented as mean ± standard deviation, absolute numbers, or median values.
*Significantly lower than cefotetan, $P \leq 0.05$.
**Significantly lower than cefotetan, $P \leq 0.01$.

TABLE 2. Types of major and minor morbidities[a]

| | Cefoxitin (N = 169) | Cefotetan (N = 168) |
|---|---|---|
| Major morbidities | | |
| Endometritis | 17 (10.1%) | 25 (14.9%) |
| Wound infection/dehiscence | 1 (0.6%) | 5 (3.0%) |
| Generalized sepsis | 2 (1.2%) | 1 (0.6%) |
| Pelvic thrombophlebitis | 2 (1.2%) | 1 (0.6%) |
| Pelvic abscess | 0 (0.0%) | 1 (0.6%) |
| Other | 0 (0.0%) | 1 (0.6%) |
| Minor morbidities | | |
| Fever | 2 (1.2%) | 4 (2.4%) |
| Wound infection | 1 (0.6%) | 5 (3.0%) |
| Uninary-tract infection | 1 (0.6%) | 3 (1.8%) |
| Other | 2 (1.2%) | 2 (1.2%) |

[a]For patients with multiple major and/or minor morbidities, each event is counted separately.

tion of labor was 12 h and median duration of ruptured membranes was 7.5 h prior to undergoing cesarean section. Patients remained in the hospital for approximately 4.5 days after surgery. There were no significant differences between the cefoxitin and cefotetan groups with respect to any of the patient characteristics described except that fewer patients underwent primary cesarean section in the cefoxitin group (63%) compared with the cefotetan group (74%). In addition, the mean number of vaginal examinations in the cefoxitin group (3.7) was lower than in the cefotetan group (4.6), and the mean parity was higher in the cefoxitin group (1.0) than in the cefotetan group (0.8). These patient characteristic relationships between the 2 study populations remained unaltered when reanalyzed for only those subjects evaluable for efficacy. The proportion of non-evaluable patients in the cefoxitin group (11%) was similar to the proportion of non-evaluable patients in the cefotetan group (10%). A total of 23 patients were entered in the study with only minor risk factors, 11 into the cetoxitin arm and 12 into the cefotetan arm.

For the evaluable patients, 87% of the patients in the cefoxitin group had no postoperative febrile morbidity compared with 79% of patients in the cefotetan group. Major morbidity occurred post-cesarean section in 11% of evaluable patients who received intraoperative cefoxitin and in 16% of evaluable patients who received intraoperative cefotetan. Included in the patients with major morbidity were 1 patient in the cefoxitin and 5 patients in the cefotetan groups who had both major and minor morbidities. Four patients in the cefoxitin group and 6 patients in the cefotetan group had multiple major morbidities. Four (2.0%) of the evaluable patients who received cefoxitin and 8 (5.0%) of those who received cefotetan developed minor morbidity only. One patient in the cefoxitin group and 2 patients in the cefotetan group had multiple minor morbidities. These differences in both major and minor morbidity between the 2 treatment groups were not statistically significant ($P = 0.14$).

Prophylactic efficacy was further examined by subpopulations of the evaluable subjects. There were no differences in morbidity between study groups for those patients having surgeries lasting >60 min, repeat cesarean section, internal-transducer monitoring, labor >18 h, or >3 vaginal examinations. However, a significant reduction in morbidity was noted in the cefoxitin group compared with the cefotetan group for those subjects having surgeries lasting <60 min ($P = 0.01$), primary cesarean section ($P = 0.03$), and having ruptured membranes ≤7 h duration ($P = 0.01$).

Table 2 lists the established etiologies for the major and minor morbidities identified in the evaluable patients in both study groups. An analysis of the data in Table 2, excluding fever alone and unclassified morbidities, demonstrated no differ-

TABLE 3. Pretherapy microbiologic results of endometrial cultures taken at the time of cesarean section

| | Cefoxitin (N = 41) | Cefotetan (N = 40) |
|---|---|---|
| *Staphylcoccus* sp. | 14 | 17 |
| *Streptococcus*, group B | 10 | 8 |
| *Gardnerella vaginalis* | 9 | 7 |
| Gamma streptococcus | 6 | 3 |
| *Lactobacillus* sp. | 5 | 5 |
| *Propionibacterium* sp. | 5 | 5 |
| *Bacteroides* sp. | 3 | 2 |
| Alpha streptococcus | 2 | 3 |
| Anaerobic gram-positive rod | 2 | 1 |
| *Peptostreptococcus* sp. | 1 | 2 |
| *Streptococcus*, group D | 1 | 1 |
| *Pseudomonas* sp. | 1 | — |
| *Corynebacterium* sp. | 1 | 4 |
| *Propiogranulosum* sp. | 1 | — |
| *Micrococcus* sp. | 1 | — |
| Yeast | 1 | 1 |
| *Enterococcus* sp. | — | 1 |
| *Proteus mirabilis* | — | 1 |
| *Escherichia coli* | — | 1 |
| *Bacteroides fragilis* | — | 1 |
| *Bacillus cereus* | — | 1 |

ence between the 2 study populations for infectious morbidity. The clinical diagnosis of endometritis accounted for 10% and 15% of the total evaluable patients in the cefoxitin and cefotetan groups, respectively. The differences noted in endometritis were not significantly different between prophylactic therapy groups.

Pretherapy intraoperative endometrial cultures were available for 94 and 86 subjects in the cefoxitin and cefotetan groups, respectively. Of these, 53 (56%) and 46 (50%) were negative for any identifiable organisms. Table 3 demonstrates the results of the culture-positive subjects for each study group. Averages of 1.5 and 1.6 organisms/culture were identified, respectively.

When major morbidity was clinically suspected, postoperative endometrial cultures were obtained by transcervical aspiration. Organisms were isolated in 10/18 evaluable patients in the cefoxitin group and 16/28 patients in the cefotetan group. Two and 3 patients in each group, respectively, were culture negative. Endometrial culture results were not available from 6 and 9 subjects, respectively, diagnosed to have major morbidity in each study group. Table 4 presents the microbiologic results of patients with major morbidity who were culture positive. Endometritis accounted for >90% of major morbidity (17/18 patients in the cefoxitin group and 25/27 patients in the cefotetan group). Polymicrobial infections were common with 2.9 and 2.7 organisms identified per culture, respectively.

TABLE 4. Endometrial isolates from patients with major morbidity[a]

| Pathogen class | Cefoxitin (N = 10) | Cefotetan (N = 16) |
|---|---|---|
| Gram-positive aerobic cocci | | |
| *Staphylococcus aureus* | 0 | 2 |
| *Staphylococcus epidermidis* | 1 | 1 |
| *Staphylococcus*, coagulase negative | 1 | 1 |
| Streptococci (group B) | 0 | 4 |
| Streptococci (group D enterococci) | 5 | 7 |
| Streptococci (group D non-enterococci) | 1 | 0 |
| Gram-negative aerobic rods | | |
| *Escherichia coli* | 3 | 0 |
| *Hemophilus influenzae* | 0 | 1 |
| *Pseudomonas aeruginosa* | 0 | 1 |
| Gram-positive anaerobic rods | | |
| *Lactobacillus* | 1 | 0 |
| Gram-positive anaerobic cocci | | |
| *Peptostreptococcus anaerobius* | 0 | 2 |
| Gram-negative anaerobic rods | | |
| *Bacteroides bivius* | 4 | 2 |
| *Bacteroides capillosus* | 1 | 0 |
| *Bacteroides melaninogenicus* | 2 | 0 |
| *Bacteroides oralis* | 0 | 1 |
| *Gardnerella vaginalis* | 0 | 1 |
| Gram-negative bacteria | | |
| Gram-negative rods | 0 | 1 |

[a]Some patients had >1 pathogen isolated. Endometritis accounted for >90% of all major morbidity.

All 375 patients enrolled in this study were included in the analysis of safety. Eight patients had a clinical adverse experience, 3 patients in the cefoxitin group and 5 patients in the cefotetan group. None of the patients in either treatment group had a serious clinical adverse experience. All clinical adverse experiences were judged to probably not, or definitely not, be related to the study drugs. The proportions of patients with clinical adverse experiences were similar in the cefoxitin and cefotetan groups. No patients were discontinued from the study due to a clinical adverse experience. Two patients in the cefoxitin group and 3 patients in the cefotetan group had a laboratory adverse experience. These differences were not statistically significant. The study drug was well tolerated in all patients in both treatment groups.

### DISCUSSION

Cesarean section is one of the most common operative procedures performed on an obstetrics and gynecology service.[5] In patients undergoing cesarean section with recognized risk factors, infectious morbidity in the form of postpartum endometritis, would infection, or urinary-tract infection is relatively frequent.[1,6,7] Numerous studies have documented the efficacy of a variety of regimens for antibiotic prophylaxis to reduce postoperative infectious morbidity.[1] Although this general approach has led to significant reductions in hospital-based costs,[8] further attempts at cost containment have prompted the recommendation to use less expensive (and less broad-spectrum) antimicrobial agents and to reduce the number of perioperative antibiotic doses.[9] A number of investigators have supported these latter recommendations, although uniform agreement does not exist due to concerns regarding confounding variables such as differences in patient population demographics.[10] Regardless, the data seem clear that absolute polymicrobial susceptibility to a given prophylactic agent is not necessary to demonstrate a reduction in infectious morbidity following cesarean section. Additionally, recent information has suggested that incipient but established infection (as opposed to bacterial colonization) at the time of surgery is a critical mechanism by which patients fail prophylaxis.[11]

By definition, antimicrobial prophylaxis is intended to reduce bacterial colonization and contamination at the time of surgery so that the host's endogenous immune responses can overcome the threat of infection.[2] Therefore, adequate tissue concentrations of a given prophylactic agent empirically should be present during the time of cesarean section. This also implies that relatively short half-life agents should be efficacious since this operative procedure uncommonly exceeds 1 h. Again, numerous studies examining such agents confirm this fact and contradict those advocating that only extended half-life agents should be used for single-dose prophylaxis.[1,6,7,9] Indeed, the data from our study, which represents one of the largest studies to compare 2 agents with a similar microbial spectrum, yet with different half-lives, support this conclusion. Of interest, similarities in efficacy held true even for those cases in which the time of surgery was extended. Although cefoxitin was found to be statistically more efficacious than cefotetan in several of our subpopulation analyses (surgery <60 min, primary cesarean section, and ruptured membranes <7 h), the clinical significance of these findings is unclear, especially since others have reported no differences between these 2 prophylactic agents.[12]

One should keep in mind the fact that hospital-related costs are most influenced by hospitalization days, intensive-care admission, and the need for additional operative procedures.[13] Therefore, antimicrobial efficacy should be the primary consideration in choosing a prophylactic agent. Given the general acceptance of single-dose prophylaxis, cost determinations should be based on gram-for-gram comparisons, not on tangential issues such as dosing intervals or nursing-administration time requirements; these are more of concern when evaluating therapeutic antibiotic regimes. Lastly, ongoing monitoring for clinical failure rates, resistance patterns, side effects, and pharmacy cost fluctuations should be undertaken at each individual institution to assure cost-effective quality care.

### REFERENCES

1. Enkin M, Enkin E, Chalmers I, Hemminki E: Prophylactic antibiotics in association with cesarean section. In Chalmers I, Enkin M, Keirse MJ (eds): Effective Care in Pregnancy and Childbirth. Oxford: University Press, pp 1246–1269, 1989.
2. Ledger WJ: Infection in the Female. Philadelphia: Lea & Febiger, pp 113–120, 1986.
3. Ward A, Richards DM: Cefotetan, a review of its antibacterial activity, pharmacokinetic properties and therapeutic use. Drugs 30:382–426, 1985.
4. Neu HC: Cefoxitin, a semi-synthetic cephamycin antibiotic: Antibacterial spectrum and resistance to hydrolysis by gram negative beta-lactamases. Antimicrob Agents Chemother 6:170–179, 1974.
5. Zahniser SC, Kendrick JS, Franks AL, Saftlas AF: Trends in obstetric operative procedures, 1980–1987. Am J Public Health 82:1340–344, 1992.
6. Swartz WH, Grolle K: The use of prophylactic antibiotics in cesarean section. J Reprod Med 26:595–609, 1981.
7. Galask RP: Changing concepts in obstetric antibiotic prophylaxis. Am J Obstet Gynecol 157:491–497, 1987.
8. Duff P: Prophylactic antibiotics for cesarean delivery: A simple cost-effective strategy for prevention of postoperative morbidity. Am J Obstet Gynecol 157:794–798, 1987.
9. Faro S, Martens MG, Hammill HA, Riddle G, Tortolero G: Antibiotic prophylaxis: Is there a difference? Am J Obstet Gynecol 162:900–909, 1990.
10. Benrubi GI: Cesarean section prophylaxis: Is anaerobic coverage necessary? South Med J 83:38040, 1990.
11. Gonik B, Shannon RL, Shawar R, Costner M, Seibel M: Why patients fail antibiotic prophylaxis at cesarean delivery: Histologic evidence for incipient infection. Obstet Gynecol 79:179–184, 1992.
12. Galask RP, Berrigno BB, Cunningham FG, Elliott JP, Makowski E, McGregor JA, Poindexter A: Results of a multicenter comparative study of single-dose cefotetan and multiple-dose cefoxitin as prophylaxis in patients undergoing cesarean section. Am J Surg 155:86–90, 1988.
13. Finkler SA: Cost Accounting for Health Care Organizations: Concepts and Applications. Gaithersburg, MD: Aspen Publishers, pp 442–444, 1994.

## References

[PDF_00472] Duff P. (1987). Prophylactic antibiotics for cesarean delivery: a simple cost-effective strategy for prevention of postoperative morbidity.. Am J Obstet Gynecol.

[PDF_00476] Faro S., Martens M. G., Hammill H. A., Riddle G., Tortolero G. (1990). Antibiotic prophylaxis: is there a difference?. Am J Obstet Gynecol.

[PDF_00487] Galask R. P., Benigno B. B., Cunningham F. G., Elliott J. P., Makowski E., McGregor J. A., Poindexter A. (1988). Results of a multicenter comparative study of single-dose cefotetan and multiple-dose cefoxitin as prophylaxis in patients undergoing cesarean section.. Am J Surg.

[PDF_00470] Galask R. P. (1987). Changing concepts in obstetric antibiotic prophylaxis.. Am J Obstet Gynecol.

[PDF_00483] Gonik B., Shannon R. L., Shawar R., Costner M., Seibel M. (1992). Why patients fail antibiotic prophylaxis at cesarean delivery: histologic evidence for incipient infection.. Obstet Gynecol.

[PDF_00461] Neu H. C. (1974). Cefoxitin, a semisynthetic cephamycin antibiotic: antibacterial spectrum and resistance to hydrolysis by gram-negative beta-lactamases.. Antimicrob Agents Chemother.

[PDF_00468] Swartz W. H., Grolle K. (1981). The use of prophylactic antibiotics in cesarean section. A review of the literature.. J Reprod Med.

[PDF_00458] Ward A., Richards D. M. (1985). Cefotetan. A review of its antibacterial activity, pharmacokinetic properties and therapeutic use.. Drugs.

[PDF_00465] Zahniser S. C., Kendrick J. S., Franks A. L., Saftlas A. F. (1992). Trends in obstetric operative procedures, 1980 to 1987.. Am J Public Health.

